# Alpha-mannosidosis in Tunisian consanguineous families: Potential involvement of variants in *GHR* and *SLC19A3* genes in the variable expressivity of cognitive impairment

**DOI:** 10.1371/journal.pone.0258202

**Published:** 2021-10-06

**Authors:** Rahma Mkaouar, Zied Riahi, Cherine Charfeddine, Imen Chelly, Hela Boudabbous, Hamza Dallali, Crystel Bonnet, Meriem Hechmi, Soumeya Bekri, Nadia Zitouna, Lotfi Zekri, Amel Tounsi, Rym Kefi, Jihene Marrakchi, Olfa Messaoud, Ichraf Kraoua, Sonia Maalej, Ilhem Turki Ben Youssef, Ahlem Ben Hmid, Fabrice Giraudet, Sami Bouchoucha, Neji Tebib, Ghazi Besbes, Christine Petit, Ridha Mrad, Sonia Abdelhak, Mediha Trabelsi

**Affiliations:** 1 Laboratory of Biomedical Genomics and Oncogenetics LR20IPT05-Pasteur Institute in Tunis, Tunis, Tunisia; 2 Department of Congenital and Hereditary Diseases, Charles Nicolle Hospital in Tunis, Tunis, Tunisia; 3 Faculty of Mathematical, Physical and Natural Sciences in Tunis, University of Tunis El Manar, Tunis, Tunisia; 4 High Institute of Biotechnology of Sidi Thabet, Biotechpole of Sidi Thabet, University of Manouba, Ariana, Tunisia; 5 Department of Paediatrics, Habib Bougatfa Hospital, Bizerte, Tunisia; 6 Faculty of Medicine in Tunis, LR99ES10 Laboratory of Human Genetics, University of Tunis El Manar, Tunis, Tunisia; 7 Department of Paediatrics and Metabolic Diseases EPS La Rabta Hospital in Tunis, Tunis, Tunisia; 8 Faculty of Medicine in Tunis, Laboratory of Hereditary Diseases of the Metabolism Investigation and Patients Management, University of Tunis El Manar, Tunis, Tunisia; 9 Faculty of Medicine in Tunis, Department of Epidemiology and Public Health, Directorate General of Military Health, University of Tunis El Manar, Tunis, Tunisia; 10 Hearing Institute, Pasteur Institute, INSERM, Paris, France; 11 National Institute of Applied Science and Technology, University of Carthage, Tunis, Tunisia; 12 Laboratory of Medical Biochemistry, Institute of Clinical Biology, University Hospital in Rouen, Rouen, France; 13 ICHARA association (International Research Institute on Sign Language), Tunis, Tunisia; 14 CNSS Polyclinic, UMA square, Bizerte, Tunisia; 15 Department of Otorhinolaryngology and Maxillofacial Surgery—La Rabta Hospital in Tunis, Tunis, Tunisia; 16 Department of Child Neurology, LR 18SP04, National Institute Mongi Ben Hmida of Neurology in Tunis, University of Tunis El Manar, Tunis, Tunisia; 17 Department of Pneumology-Abderahman Mami Hospital in Ariana, Ariana, Tunisia; 18 Laboratory of clinical immunology, Pasteur Institute in Tunis, Tunis, Tunisia; 19 Faculty of Medicine, Neurosensory Biophysics, INSERM 1107, University of Clermont Auvergne, Clermont-Ferrand, France; 20 Department of Paediatric Orthopaedics and Traumatology -Children’s Hospital Bechir Hamza, Tunis, Tunisia; 21 College of France, Paris, France; University of Iowa, UNITED STATES

## Abstract

Alpha-Mannosidosis (AM) is an ultra-rare storage disorder caused by a deficiency of lysosomal alpha-mannosidase encoded by the *MAN2B1* gene. Clinical presentation of AM includes mental retardation, recurrent infections, hearing loss, dysmorphic features, and motor dysfunctions. AM has never been reported in Tunisia. We report here the clinical and genetic study of six patients from two Tunisian families with AM. The AM diagnosis was confirmed by an enzymatic activity assay. Genetic investigation was conducted by Sanger sequencing of the mutational hotspots for the first family and by ES analysis for the second one. In the first family, a frameshift duplication p.(Ser802GlnfsTer129) was identified in the *MAN2B1* gene. For the second family, ES analysis led to the identification of a missense mutation p.(Arg229Trp) in the *MAN2B1* gene in four affected family members. The p.(Ser802GlnfsTer129) mutation induces a premature termination codon which may trigger RNA degradation by the NMD system. The decrease in the levels of MAN2B1 synthesis could explain the severe phenotype observed in the index case. According to the literature, the p.(Arg229Trp) missense variant does not have an impact on MAN2B1 maturation and transportation, which correlates with a moderate clinical sub-type. To explain the intra-familial variability of cognitive impairment, exome analysis allowed the identification of two likely pathogenic variants in *GHR* and *SLC19A3* genes potentially associated to cognitive decline. The present study raises awareness about underdiagnosis of AM in the region that deprives patients from accessing adequate care. Indeed, early diagnosis is critical in order to prevent disease progression and to propose enzyme replacement therapy.

## Introduction

Alpha-Mannosidosis (AM; OMIM #248500) is an ultra-rare autosomal recessive storage disorder induced by the deficiency of lysosomal Alpha-Mannosidase (LAMAN, EC 3.3. 1.24) [1]. Despite its rarity, the disease is globally widespread; its prevalence has been estimated to 1/1.000.000 people in the general population [2]. The main clinical manifestations of AM include intellectual deficiency (ID), immunodeficiency, hearing loss, dysmorphic features, skeletal abnormalities, psychiatric disorders, dysostosis, and motor dysfunctions [3].

AM is characterized by a progressive disease course and has been classified into three clinical sub-types based on disease severity and age at onset. Type 1 is the mildest form with slow progression of mental retardation, no skeletal abnormalities, and is usually diagnosed during or after adolescence (>10 years old). Type 2 form is relatively moderate; it begins before the age of 10 and is distinguished by skeletal abnormalities, slow progression of ID, and the possibility of developing ataxia by the age of 20–30 years. Type III is the most severe form of the disease diagnosed in the neonatal period with rapid progression followed by death due to alteration of the central nervous system [4,5].

Alpha-Mannosidase is an acidic exoglycosidase involved in the hydrolysis of mannosidic linkages during the degradation of N-linked oligosaccharides [6–8]. Therefore, affected cells accumulate undigested oligosaccharides in large vacuoles in the lysosomes. Lysosomal saturation by storage material hampers several cellular functions such as synaptic release, Ca^2+^ influx, and apoptosis. Nevertheless, physiopathological pathways have not been fully elucidated [9].

AM is caused by mutations in the *MAN2B1* gene (* 609458, NM_000528.4) located at chromosome 19p13.2 and comprising 24 exons [10]. This gene encodes the α-Mannosidase lysosomal enzyme, which is synthesized as a polypeptide of 1011 amino acids and is post-translationally modified in the endoplasmic reticulum [8,11].

During the maturation and transport of LAMAN to the lysosomes, the enzyme is proteolytically cleaved starting from the N-terminus to produce three major polypeptides referred to as peptides ‘abc’, ‘d’, and ‘e’ [8,9].

The mutational spectrum of AM includes different types of variants: missense mutations (32%), nonsense mutations (19%), splice site mutations (15%), small deletions (14%), small duplications (13%), small insertions (2%), and large deletions (2%). To date, 178 variants have been identified in 218 patients from 41 countries (https://apex.jupiter.no/apex/f?p=101:1). Reported patients mostly originate from Europe, with only 13 patients from the Arab world including Algeria, Morocco, Palestine, United Arab Emirates, Kuwait, and Saudi Arabia [12].

To the best of our knowledge, clinical and genetic investigations of AM have never been carried out on Tunisian patients. The Alpha-mannosidosis Mutation Database referred to one Tunisian case for which the clinical and genetic data are not available (https://apex.jupiter.no/apex/f?p=101:1).

The current work reports the first clinical and genetic study of AM in Tunisia.

## Materials and methods

Written informed consent was obtained from all patients or their legal guardians for the minors. The present study was approved by the Pasteur Institute in Tunis Review Board (Reference Number:2017/34/E/HRT) and conducted according to the ethical principles defined by the Declaration of Helsinki.

### Patient recruitment

Two Tunisian families (TNDF617 and TNDF182), including six affected members, were recruited at the Oto-Rhino-Laryngology Department at La Rabta Hospital in Tunis and the Congenital and Hereditary Diseases Department at Charles Nicolle Hospital in Tunis. The two families originate from Northern Tunisia. Consenting and available family members were interviewed following a questionnaire that allowed the collection of data on genealogy, geographical origin, history of miscarriages and neonatal deaths, course of pregnancy, perinatal suffering as well as family history of diseases.

### Methods

#### Clinical evaluation

A multidisciplinary clinical investigation was performed considering the multisystemic impairment caused by the disease. Affected individuals underwent a comprehensive clinical evaluation that included ocular fundus examination, cardiac, and renal echography, pulmonary examination, dysmorphology, cognitive, and neurological assessments. Audiological evaluation was based on the Brainstem Evoked Response (BER) test. A range of subjective hearing examinations were also conducted including pure-tone audiometry, speech audiometry (intelligibility tests), and reflex audiometry (acoustic reflex thresholds, visual reinforcement, play audiometry).

#### Alpha-mannosidase activity assay

Alpha-mannosidase activity was measured in leukocytes from fresh peripheral blood by density gradient separation using Histopaque-1 077 (Sigma-Aldrich, St. Louis, Missouri). Cell lysate was incubated in 4 mM substrate in 0.1 M acetic acid (pH 4.5) at 37°C for 1–3 hours. Lowry method was used to determine the protein concentration using bovine albumin as a standard. The fluorometric assay was performed using the artificial substrate 4- ethylumbelliferyl alpha-D-mannopyranoside (Sigma-Aldrich) at pH 4 (0.1 M sodium acetate buffer) and 37°C. The reaction was terminated by the addition of a solution containing 133 mM glycine, 67 mM NaCl, 83 mM Na2CO3 (pH 10.7) at an equal volume [8,13].

#### Exome Sequencing (ES)

Genomic DNA was extracted from peripheral blood leucocytes using standard protocols. Exon capture was performed using the Agilent SureSelect XT Human All Exon v6 (60 Mb) Version C2 (Dec 2018). After library preparation, samples were sequenced on a NovaSeq6000 platform (Illumina, San Diego, California) with a 150 bp paired-end reads configuration. Raw sequence files were aligned to the human genome reference sequence (version GRCh38) using the Burrows-Wheeler Aligner-MEM (BWA-MEM version 1.1.1; https://bio-bwa.sourceforge.net). Duplicates were removed from BAM files using PICARD tool (www.picard.sorceforge.net). The Genome Analysis Tool Kit (GATK, www.broadinstitute.org/gatk/) was used for Indels realignment by the Realigner Target Creator tool. GATK was also used for base quality score recalibration. Variant Calling was performed by the GATK package Haplotype Caller.

Genetic variants were annotated and prioritized using the VarAFT software version 2.16 (http://varaft.eu/). SNPs and INDELs located in functionally relevant genomic regions (exonic, splicing) were selected. Variants identified as synonymous or non-coding were filtered out. Sequencing coverage statistics were calculated using the VarAFT tool. Genetic variants, with a read depth<20X and a mapping quality score<30, were discarded. On the assumption that significant disease-causing mutations are rare, a frequency filter was applied to only include variants with a Minor Allele Frequency (MAF) threshold of 0.1 according to 1000Genomes (https://www.internationalgenome.org/), Exac (http://exac.broadinstitute.org), GnomeAD(https://gnomad.broadinstitute.org/), and GME (Greater Middle East) Variome (http://igm.ucsd.edu/gme/) databases.

In the subsequent set of variants with a MAF<0.1, we selected those located in both non syndromic (238) and syndromic (70) deafness genes by referring to two specific hearing loss databases: Hereditary Hearing Loss Homepage (HHLH, https://hereditaryhearingloss.org/) and Deafness Variation Database (DVD, http://deafnessvariationdatabase.org/) as well as the general database Online Mendelian Inheritance in Men (OMIM, https://www.omim.org/). We have also targeted genes involved in sensorineural signalling pathways according to the Kyoto Encyclopaedia of Genes and Genomes database (KEGG, https://www.genome.jp/kegg/).

The functional effects of genetic sequence variants were evaluated by *in silico* prediction tools including SIFT (sift.bii.a-star.edu.sg/), MutationTaster (mutationtaster.org/); PolyPhen (genetics.bwh.harvard), FATHMM (http://fathmm.biocompute.org.uk), PROVEAN v1.1 (provean.jcvi.org/), MutationAssessor 1.0 (mutationassessor.org/r3/), and VarSome (https://varsome.com). Genetic variants, for which the CADD-Phred score is less than 15, were filtered out.

#### Copy number variation (CNV) detection and analysis

CNVs were called from ES data of the index case TNDF182-3 using the ExomeDepth R package that detects CNVs from exome experiments based on a read depth approach. The tested exome was compared to a matched aggregate reference set that combines five control exomes generated by identical NGS sequencing and bioinformatic analysis procedures.

#### Filtering strategy and identification of pathogenic variants in TNDF182-6 patient

The aim of the ES analysis performed in TNDF182-6 patient was to identify variants of clinical significance related to cognitive impairment, in addition to the *MAN2B1* gene mutation. Indeed, we consulted the OMIM database to extract gene lists associated to four disease categories: neuropathology (182), ID (185), cognitive impairment (187), and metabolic diseases (181). We also carried out a variant screening in the genes involved in *MAN2B1* gene (NM_000528.4) biological pathways and network interactions according to GeneMania prediction web server (https://genemania.org/), stringdb 11.0 (https://string-db.org/), and Phenolyzer (https://phenolyzer.wglab.org/) databases. The public databases Reactome (https://reactome.org/), UniprotKB (https://www.uniprot.org/uniprot/), GO central (http://geneontology.org/), and Genecards (https://www.genecards.org/) were used to retrieve the biological pathways implicating the selected genes of interest. The multiple genomic alignments tool (https://www.ensembl.org/Homo_sapiens/Gene/Compara_Alignments) in Ensembl genome browser (https://www.ensembl.org/) was used to align homologous nucleotide sequences to human *SLC19A3* and *GHR* genes. The UniprotKB database (https://www.uniprot.org/align/) was also used to align homologous protein sequences to human SLC19A3 and GHR proteins with the Clustal omega tool. The degree of conservation was retrieved from the Conserved Domain Database (CDD, https://www.ncbi.nlm.nih.gov/cdd).

#### Sanger sequencing

Exons 14, 18, and 20 of the *MAN2B1* gene (NM_000528.4) were screened by Sanger sequencing in order to identify the genetic aetiology of AM in the first index case TNDF617-1. Sanger sequencing was performed for the index case (TNDF182-3) to screen for variants in the three most frequent deafness related genes in the Tunisian population: namely *GJB2* (**#** 220290, NM_004004.6), *SLC26A4* (* 605646, NM_000441.2), and *TMC1* (* 606706, NM_138691.2) genes.

Following ES analysis, Sanger sequencing was used to validate the selected variants in *MAN2B1*, *GHR* (* 600946, NM_000163.5), and *SLC19A3* (*606152, NM_025243.4) genes in the probands and to check the familial segregation when applicable. It was also used to perform cascade screening of the disease- causing mutation identified in exon 5 of the *MAN2B1* gene in all affected members of TNDF182 family.

Polymerase Chain Reaction (PCR) was performed on genomic DNA samples using primers (available upon request) designed by the primer3 (http://primer3.ut.ee) software. PCR products were sequenced with the Big Dye terminator v3.1 cycle sequencing reaction kit on an ABI prism 3130 DNA Genetic Analyzer (Applied Biosystems, Foster City, CA, USA) in accordance with the manufacturer’s recommendations.

## Results

### Clinical features

The clinical characteristics of the six affected Tunisian cases are summarized in Table 1.

**Table 1:** Clinical features of all investigated cases.

Family ID	TNDF182					TNDF617	
Patient ID	TNDF1823	TNDF1825	TNDF1826	TNDF1827	TNDF1828	TNDF6171	TNDF6172
Sex	F	F	F	F	M	M	F
Age	9	3	7	18	33	19	Deceased at 16 years
Age at diagnosis of AM (years)	8	2	6	17	33	18	4
Age at onset of Hearing impairment (years)	18 months	9 months	ND†	10 months	2	ND	ND
Degree of Hearing impairment	Moderately severe progressive bilateral (68-72db for frequencies between 0.25 and 8 kHz)	Moderate bilateral (50-54db) for frequencies between 0.25 and 8 kHz)	Progressive bilateral (50-60db), psychomotor delay	Severe to profound bilateral (80–100 dB)	ND	ND	ND
Dysmorphic features:	Narrow and Protruding forehead, slight retrognathism, Bulbous nasal tip Low-set left ear Gum hypertrophy	Midface hypoplasia, Frontal bone hypoplasia, synophris, frontal swellings, depressed nasal bridge, Bulbous nasal tip Large protruding ears	Coarse facial features, slight retrognathism, Midface hypoplasia protruding, bushy and arched eyebrows bulbous nasal tip, thick alae nasi, long philtrum, Slightly protruding ears Small set of teeth with low density enamel	Large and broad forehead, bi-temporal depression, slight retrognathism, Midface hypoplasia bulging eyes, bushy eyebrows, asymmetric eyeballs with retraction of the lower left eyelid depressed nasal root, tucked-up nose tip with anteverted nostrils, short philtrum, fleshy lips	Coarse facial features, midface hypoplasia	Coarse facial traits	Coarse facial features
Musculoskeletal abnormalities **Skeleton X-ray**	Pectus carinatum, spondylolisthesis (L5/S1)	Lumbar kyphosis	Lumbar kyphosis, pectus carinatum, Short neck	Pectus carinatum, lumbar kyphosis, dorso-lumbar scoliosis, hyper-lordosis, platyspondyly of the dorsal vertebrae, short spinous processes, short neck, gibbosity on the right side, cubitus valgus	Thoracic distortion, scoliosis, lower limbs deformities	Thoracic distortions: scoliosis all along the lower vertebrae, pectus excavatum	Skeletal malformations, bone remodelling defect
Neurological manifestations **Cerebral MRI** **Neuromuscular/Motor abnormalities**	--	--	Microcephaly	Muscular fatigability	-- Motor disability (uses a wheelchair)	Global developmental delay Microcephaly Trigonocephaly Cerebral palsy Encephalopathy Periventricular leukomalacia Spastic tetraparesis	Psychomotor delay Craniostenosis Cerebral palsy Spastic tetraparesis
Cognitive decline	Learning difficulties	--	Intellectual disability, Autism spectrum disorder traits (Stereotypic behaviour)	Memory impairment, learning difficulties	Mental retardation	Mental retardation (Moderate)	Mental retardation
Psychiatric symptoms **Behaviour**	--	--	Hyperactivity	--	Hetero-aggressive behaviour	--	--
Immunodeficiency **Immunoglobulins assay** **(IgG, IgA, IgM)**	Recurrent seromucosal otitis treated with tympanostomy tubes, Purulent otorrhea, erythematous angina, recurrent nasopharyngeal infections Normal	Recurrent Broncho-pulmonary infections Normal	Recurrent Broncho-pneumopathies since the age of 2 months, adenoids hypertrophy ND	Adenoid hypertrophy ND	-- ND	-- ND	-- ND
Ocular Examination	Hypermetropy, strabismus	--	--	High Myopia	Strabismus	Convergent Strabismus, bilateral corneal arcus	--
Abdominal sonography	Hepatomegaly	Hepatosplenomegaly	--	--	--	Splenomegaly	Hepatosplenomegaly (Moderate)
Other features	Mongolian blue spots on the back Hirsutism on the back and limbs	Mongolian blue spots on the back Obstructive Sleep apnoea	Hirsutism Noisy breathing Nasal voice	--	--	Haematuria, Mongolian blue spots, café au lait skin patches	--
Enzymatic activity (pmol/punch/h)	0	0	ND	ND	ND	ND	0

Table 1 summarizes the results of the clinical investigation of all the patients enrolled in the current study.

†Not Determined.

### TNDF617 family

**Fig 1:**
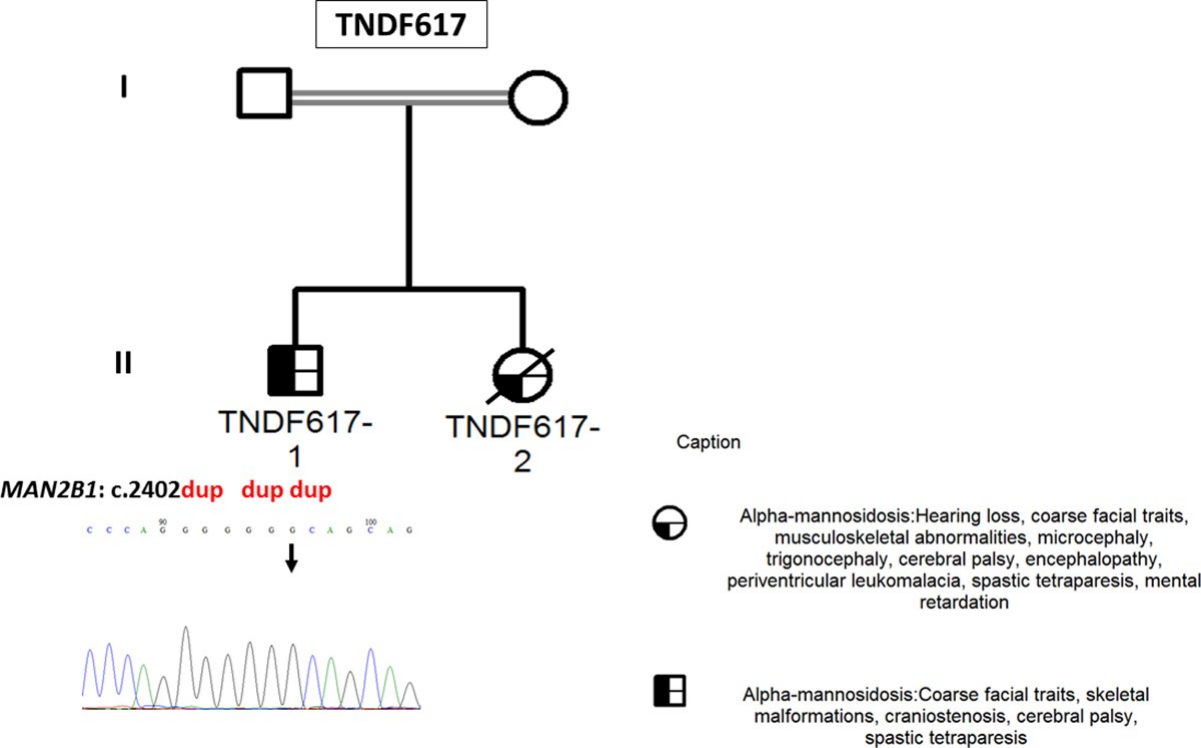
TNDF617 Family pedigree and chromatogram of the disease-causing variant identified in the *MAN2B1* gene. The arrows indicate the cases for whom the molecular diagnosis was performed. Double lines indicate consanguinity. Red and bold denote the *MAN2B1* gene (NM_000528.4) variant c.2402dup carried by the index case (TNDF617-1).

This family originates from a city located in North-Eastern Tunisia. The first individual diagnosed with AM is the index case’s sister (Individual TNDF617-2, Fig 1) in 2003 at the National Institute of Neurology Mongi BEN HMIDA in Tunis. She was examined by a paediatric neurologist for craniostenosis, cerebral palsy, and mental retardation. In the presence of coarse facial features associated with skeletal malformations, clinicians have made the diagnosis of a lysosomal storage disease that was suspected at first to be a mucopolysaccharidosis. Alpha-mannosidase activity assay, carried out in this patient (TNDF617-2), revealed a null enzymatic activity. She passed away, in 2015, at the age of 16 years old.

At the time of diagnosis, TNDF617-2’s mother was pregnant with the index case (TNDF617-1) and was addressed for prenatal diagnosis. Unfortunately, she never showed up for amniocentesis. At the age of 15, the index case (TNDF617-1) was addressed for genetic investigation in order to determine the molecular aetiology of spastic tetraparesis that had developed over the course of four years. Taking into consideration his familial history, a diagnosis of AM was suspected. TNDF617-1 patient presented with ID, deafness, coarse facial features, and skeletal malformations.

### TNDF182 family

**Fig 2:**
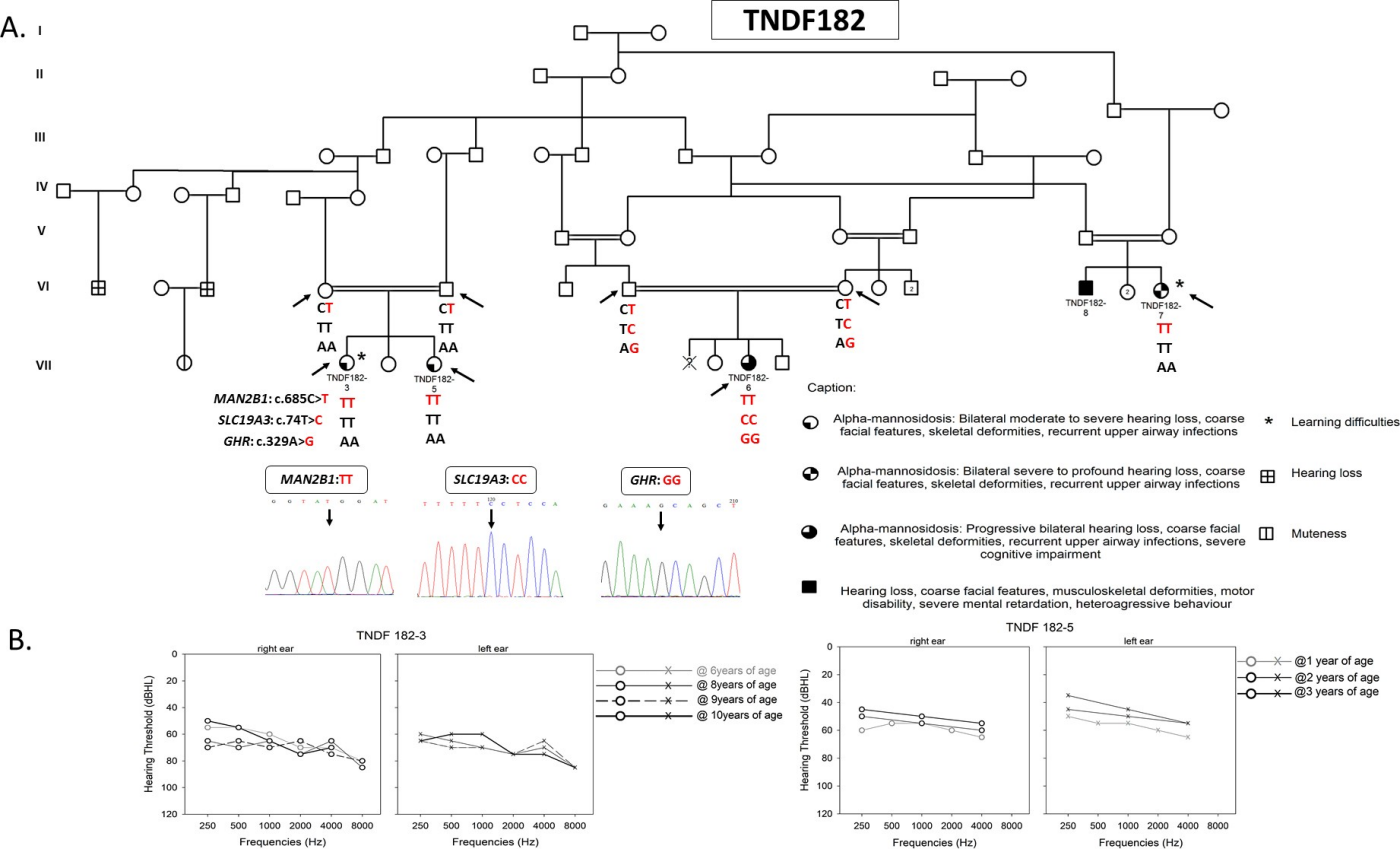
TNDF182 family pedigree and audiometric data from TNDF182-3 and TNDF182-5 patients. The extended TNDF182 family. The arrows indicate the cases for whom the molecular diagnosis was performed. Double lines indicate consanguinity. Red and bold denote the mutant alleles for the three variants identified in *MAN2B1* (NM_000528.4), *GHR* (* 600946, NM_000163.5), and *SLC19A3* (*606152, NM_025243.4) genes. Chromatograms of each variant are shown (A). Audiograms of patients TNDF182-3 and TNDF182-5 were obtained using pure-tone audiometry (frequencies from 250 Hz to 8,000 Hz) performed at different ages (B).

This family originates from a town located in the North-Eastern region of Tunisia. TNDF182 is a large consanguineous family in which four affected members were identified in three different familial branches (Fig 2). All patients were born to first or second-degree consanguineous parents. The index case (TNDF182-3) was initially diagnosed with hearing impairment (HI) associated to dysmorphic features corresponding to an undefined clinical entity. Later on, her younger sister (TNDF182-5) was also diagnosed with a similar clinical presentation. Communication of the genetic results to the parents encouraged them to spread the information among other family members, at large. This allowed cascade screening of two other cousins presenting a similar phenotype. We were unable to perform a genetic test for a third cousin (TNDF182-8) due to his heavy disability and inability to give his assent. Alpha-mannosidase activity assay showed null enzymatic activity for the index case TNDF182-3 and her sister TNDF182-5.

HI ranging from moderate to profound, was diagnosed in all cases (Table 1). The index case (TNDF182-3) was diagnosed with a moderately severe HI (68–72 dB). Speech audiometry tests revealed that the patient’s auditory threshold is at the limit of conversational voice. The patient had difficulties to pronounce certain words. Learning difficulties were also noted by the parents. For her sister TNDF182-5, pure tone audiometry test revealed a moderate hearing loss (50-54db). After 3 years of follow-up, HI evaluation showed fluctuations that are likely related to recurrent otitis causing conductive hearing loss. The progression of HI in TNDF182-3 and 5 was evaluated on the basis of audiograms performed at different ages, which showed that their deafness seems to remain stable over time (Fig 2).

Dysmorphic features varied between patients; however, coarse facial features were commonly present. Radiological skeleton examination showed musculoskeletal abnormalities including pectus carinatum and lumbar kyphosis in three patients (Table 1). All four cases of the TNDF182 family shared a history of recurrent infections of the upper respiratory tract.

Among the TNDF182 family cases, TNDF182-6 is distinguished by a more severe cognitive impairment manifested by ID and stereotypic behavioural traits of autistic spectrum disorder. As for the cognitive decline in her cousins (TNDF182-3 and TNDF182-7), intellectual disability was observed. The patient (TNDF182-6) regularly attends speech behavioural and psychomotor therapy sessions. She went to kindergarten and currently attends primary school accompanied by a personal assistant. She did not accept to wear hearing aids because of her hyperactive behaviour.

### Genetic investigation

#### TNDF617 family

Considering the family history of AM in the deceased sister, we performed a mutational screening of the *MAN2B1* gene by targeting in priority the founder mutation c.2248C>T; p.(Arg750Trp) reported in patients originating from Europe and Turkey as well as two recurrent mutations: c.1830+1G>C, and c.2426T>C; p.(Leu809Pro), located in intron 14, exon 18, and exon 20, respectively. These are the three most common variants reported in AM patients, accounting for 35.4% of disease-causing alleles [8]. None of these variants were found in the index case (TNDF617-1), however, a homozygous frameshift duplication, c.2402dup; p.(Ser802GlnfsTer129), was identified in exon 20 of the *MAN2B1* gene.

According to GnomAD, this variant has a MAF of 3.979е-6 in the European (Non-Finnish) population. It was not found in our in-house control database (92 exomes), nor in the other sample populations represented in GnomAD database and GME Variome Project (S1 Table).

#### TNDF182 family

The clinical presentation of the index case was firstly suspected as a hereditary HI fortuitously associated to skeletal malformations. After exclusion of the three most frequently mutated deafness genes in Tunisia: *GJB2*, *TMC1*, and *SLC26A4* genes, ES was carried out for the index case TNDF182-3.

ES results were interpreted following the variant prioritization steps described above. Clinically relevant variants were extracted by targeting more than 500 genes involved in non-syndromic and syndromic deafness as well as diseases including HI. Thirty-five variants were detected in 21 hearing loss related genes corresponding to 11 VUSs, 22 benign, and 2 pathogenic variants located in *OTOG* (*604487, NM_001277269) and *MAN2B1* genes (S2 Table). For the *OTOG* gene, it is a heterozygous stop gain variant c.8726G>A; p.(Trp2909Ter) in exon 55. The presence of a CNV as a second pathogenic allele was ruled out. Considering that disease causing variants in the *OTOG* gene are known to segregate according to an autosomal recessive pattern, the presence of a monoallelic non-sense mutation led us to exclude its implication in HI in the index case. The second variant found in exon 5 of the *MAN2B1* gene is a homozygous missense variant, c.685C>T; p.(Arg229Trp), that was previously reported as disease-causing in populations originating from Northern and Eastern-Europe, USA, and Jamaica (Table 2) [8]. According to GnomAD, this mutation has been uniquely found in the European population (Non-Finnish and Finnish) genomes with a global MAF of 1.314е-5. It was not found in the exome data of our local control database (S1 Table).

**Table 2:** Mutational, phenotypic, and geographical data of patients carrying the *MAN2B1* (NM_000528.4) gene disease-causing variants identified in TNDF182 and TNDF617 families.

Country of origin	Number of patients	Clinical sub-type	Nucleotide change (genomic level, genome build GRCh38.p13)	Nucleotide change (cDNA level)	Amino acid change	References
**New Zealand**	**1**	**Type 2**	**chr19:12,663,781G>A/chr19:12,655,693C>G**	**c.685C>T/c.1830+1G>C**	**p.(Arg229Trp)/p.(Val549_Glu610del)**	**[8];** **(https://apex.jupiter.no/apex/f?p=101:1)**
**New Zealand**	**1**	**Type 2**	**chr19:12,663,781G>A/chr19:12,655,693C>G**	**c.685C>T/c.1830+1G>C**	**p.(Arg229Trp)/p.(Val549_Glu610del)**	
**USA**	**1**	**Type 2**	**chr19:12,663,781G>A/chr19:12,649,932G>A**	**c.685C>T/c.2248C>T**	**p.(Arg229Trp)/p.(Arg750Trp)**	
**Jamaica**	**1**	**Type 2**	**chr19:12,663,781G>A/chr19:12,652,270C>T**	**c.685C>T/c.1929G>A**	**p.(Arg229Trp)/p.(Trp643Ter)**	
**United Kingdom**	**1**	**Type 1**	**chr19:12,663,781G>A/chr19:12,648,395->CGGTGC**	**c.685C>T/c.2439_2444dupGCACCG**	**p.(Arg229Trp)/p(.His814_Arg815dup)**	
**Tunisia**	**4**	**Type 2**	**chr19:12,663,781G>A/chr19:12,663,781G>A**	**c.685C>T/c.685C>T**	**p.(Arg229Trp)/p.(Arg229Trp)**	**Present study**
**Kuwait**	**1**	**Type 3**	**chr19:12,649,170->C/chr19:12,649,170->C**	**c.2402dup/c.2402dup**	**p.(Ser802GlnfsTer129)/p.(Ser802GlnfsTer129)**	**[8];** **(https://apex.jupiter.no/apex/f?p=101:1)**
**Unknown**	**1**	**Type 2 or 3**	**chr19:12,649,170->C/chr19:12,649,170->C**	**c.2402dup/c.2402dup**	**p.(Ser802GlnfsTer129)/p.(Ser802GlnfsTer129)**	
**Lithuania**	**1**	**Type 2**	**chr19:12,649,170->C/chr19:12,664,861C>-**	**c.2402dup/c.561delG**	**p.(Ser802GlnfsTer129)/p.(Arg188AspfsTer58)**	
**United Kingdom**	**1**	**Type 2**	**chr19:12,649,170->C/chr19:12,657,482G>T**	**c.2402dup/c.1383C>A**	**p.(Ser802GlnfsTer129)/p.(Tyr461Ter)**	
**United Kingdom**	**1**	**Type 2**	**chr19:12,649,170->C/chr19:12,657,482G>T**	**c.2402dup/c.1383C>A**	**p.(Ser802GlnfsTer129)/p.(Tyr461Ter)**	
**Tunisia**	**1**	**Type 2**	**chr19:12,649,170->C/chr19:12,649,170->C**	**c.2402dup/c.2402dup**	**p.(Ser802GlnfsTer129)/(Ser802GlnfsTer129)**	**Present study**

Cascade screening targeting the *MAN2B1* mutation p.(Arg229Trp) was then performed by Sanger sequencing for the index case’s sister (TNDF182-5) and her two cousins (TNDF182-6 and TNDF182-7). The mutation was present at a heterozygous state in both parents of the index case (TNDF182-3) and in those of her cousin TNDF182-6 (Fig 2).

As TNDF182-6 patient presented with an early onset of severe ID associated with autistic traits, we hypothesized that she might carry genetic alterations in disease loci involved in cognitive impairment.

A total of 735 genes involved in all diseases and syndromes associated with ID and cognitive impairment were analysed. A total of 403 variants (Table 3) have been identified in the prioritized genes (S3 Table) among which 64 variants were rare. Two likely-pathogenic variants c.329A>G; p.(Asn110Ser) and c.74T>C; p.(Phe25Ser) were identified in *GHR* and *SLC19A3* genes, respectively (Table 3). According to UniprotKB, Reactome, GO central, and GeneCards databases, both genes are involved in biological pathways that contribute to the development of the CNS (S4 Table).

**Table 3:** Variant prioritization workflow in the exome analysis of TNDF182-6 patient.

Total number of variants	97,415
Number of functionally relevant variants (Exonic, splicing, 3’UTR, 5’UTR, upstream, downstream)	**20,500**
Number of variants with a MAF<0,1 (1000Genomes, Exac, GnomAD)	**3,297**
Number of Deleterious Variants (SIFT, POLYPHEN, POLYPHEN2, LRT, Mutation Taster, Mutation Assessor, FATHMM, PROVEAN, MetaSVM, MetaLR, M-CAP)	**1516**
Number of prioritized genes (OMIM)	**735**
Cognitive impairment/mental retardation	**372**
Neuropathies	**182**
Metabolic Diseases	**181**
Number of variants in prioritized genes	**403**
Number of variants of interest	**2:** ***SLC19A3* (NM_025243): exon2: chr2:227,702,245A>G; c.74T>C/c.74T>C; p.(Phe25Ser)/p.(Phe25Ser)** ***GHR* (NM_001242406): exon4: chr5:42,694,979A>G; c.329A>G/c.329A>G; p.(Asn110Ser)/p.(Asn110Ser)**

Sanger validation of these two variants and evaluation of the familial segregation have been carried out; both variants were present at a homozygous state in TNDF182-6 patient. The parents were heterozygous for these variants and the other affected cousins had the WT alleles (Fig 2, S1 File).

The two variants p.(Phe25Ser) and p.(Asn110Ser) respectively found in *SLC19A3* and *GHR* genes were predicted as likely pathogenic according to VarSome tool whose classification was based on four ACMG rules PM1, PM2, PP2, and PP3 [14]. Both variants are located in mutational hotspots in a region of 25 base-pairs on either side of the variants. These mutational hotspots are within functional protein domains corresponding to the transmembrane helical domain of the thiamine transporter 2 (ThTr-2) and the extracellular domain (TOPO_DOM 19–264) of the Growth hormone receptor, encoded by *SLC19A3* and *GHR* genes, respectively. The ratios of pathogenic variants in both domains are equal to 100% (greater than the 50% threshold). These arguments align with the ACMG rule PM1. Both variants were not found in 1000 Genomes Project, GnomAD, as well as the in-house local control database (Pathogenicity criteria PM2). In the *SLC19A3* gene, 22 out of 34 missense variants are pathogenic (64.7% which is more than the 51% threshold). Similarly, in the *GHR* gene, 32 out of 44 missense variants are pathogenic (72.7% which is greater than the 51% threshold). These data support the ACMG rule PP2.

The variant in the *SLC19A3* gene has been predicted as deleterious by ten *in silico* prediction algorithms including FATHMM-MKL, LRT, MetaLR, MetaSVM, MutationAssessor, Mutation Taster, PROVEAN, SIFT, Polyphen2, M-CAP, and MutPred. The p.(Asn110Ser) mutation identified in the *GHR* gene has been predicted as damaging by eight prediction tools, namely FATHMM-MKL, LRT, MetaLR, MetaSVM, Mutation Taster, Polyphen2, M-CAP, and SIFT versus two benign predictions from PROVEAN and FATHMM (ACMG category PP3). In addition, the multiple protein sequence alignments between nine different species (S2 File) showed that the Asn110 and the Phe25 residues are highly conserved (PP3). Moreover, the segregation data (PP1) showing the absence of the two variants in other healthy and affected relatives in the extended family as well as their heterozygous state in the patient’s parents further support their pathogenicity.

## Discussion

We report here clinical and genetic investigations of AM in Tunisian families. The clinical presentation of the disease varied widely between the reported families and cases particularly in regard to neurological signs.

The index case TNDF617-1 developed neurological features during infancy, including microcephaly and cerebral palsy. He also suffers from spastic tetraparesis that appeared at the age of 15 years. Cerebral palsy and spastic tetraparesis were also seen in his deceased sister (TNDF617-2). A large variety of neurological and neuromuscular manifestations have been described in the literature [4,13,15]. However, microcephaly, cerebral palsy, and spastic tetraparesis observed in TNDF617 family have not been reported so far. Mongoloid skin spots and corneal arcus (CA) observed in patients TNDF617-1, TNDF182-3, and TNDF182-5, are two other clinical features described for the first time in association with AM. CA is an ocular anomaly caused by the accumulation of lipidic deposits in the peripheral cornea. This feature is often secondary to a normal aging process and is usually seen in elderly patients. However, a strong correlation between lipid metabolism deficit, and the development of CA has also been established [16]. Other corneal anomalies have been described in AM patients, including corneal haze, corneal clouding, and corneal opacities [15,17]. Whether CA is caused by AM or due to the co-occurrence of AM and a lipid metabolism impairment needs to be further elucidated.

The genetic test in the index case TNDF617-1 was based on direct sequencing by targeting the *MAN2B1* gene mutational hotspots encompassing the founder mutation c.2248C>T and the two recurrent variants c.1830+1G>C, and c.2426T>C located in exon 18, intron 14, and exon 20, respectively. TNDF617-1 did not carry any of these mutations, instead, a homozygous frameshift duplication p.(Ser802GlnfsTer129) was identified in exon 20 of the *MAN2B1* gene. This variant was previously described by *Stensland et al*. in five patients originating from Lithuania, United Kingdom, and Kuwait (Table 2) [8]. We hypothesize that the p.(Ser802GlnfsTer129) mutation does not allow to retain residual WT protein activity considering that the enzymatic assay revealed a null enzymatic activity in the affected sibling (TNDF617-2). The p.(Ser802GlnfsTer129) truncating mutation induces a premature termination codon which may alter the stability of the mRNA and trigger its degradation by the NMD (Nonsense Mediated mRNA Decay) system [18]. Indeed, it has been reported for other monogenic disorders such as Tay-Sachs disease and Duchenne muscular dystrophy that a considerable reduction of protein biosynthesis lead to a severe phenotype [19]. We could not further explore this hypothesis due to the unavailability of RNA for this patient (TNDF617-1).

For the second family TNDF182, we identified a previously described mutation, p.(Arg229Trp), in exon 5 of the *MAN2B1* gene (Table 2). This variant was considered to be better tolerated than other disease-causing mutations as it allows the protein to be lysosomally transported [20]. Study of the impact of the p.(Arg229Trp) variant on the protein structure has shown that this mutation does not radically impact the residue volume of the protein nor drastically alter its three-dimensional conformation. Enzymatic activity measured in transfected cells have demonstrated that the p.(Arg229Trp) missense variant allows for maintaining 20–30% of the WT enzymatic activity [8]. However, enzymatic activity assays carried out for TNDF182-3 and TNDF182-5 patients revealed a null alpha-mannosidase activity despite presenting the intermediate clinical sub-type 2. This is not surprising as previous studies showed a non-significant phenotype/genotype correlation in AM patients [9].

An intrafamilial variable expressivity was observed among the second familial cases (TNDF182). This is illustrated by the faster disease progression observed in patient TNDF182-6, who manifested an earlier onset of cognitive impairment, although she is carrying the same *MAN2B1* gene mutation. Based on previous studies that showed co-occurrence of two or several diseases in the same individual, we suspected that other genes’ defects might be responsible for the cognitive deficit. To check this hypothesis, we have performed an ES analysis for this patient.

Targeting genes implicated in both isolated and syndromic forms of cognitive impairment has allowed us to select two variants of interest in *GHR* and *SLC19A3* genes. The novel missense mutation p.(Phe25Ser) in the *GHR* gene was found at a homozygous state in TNDF182-6 patient. Her parents were heterozygous and her affected cousins (TNDF182-3, 5 and 7) did not carry this variant.

Homozygous mutations in the *GHR* gene have been associated with Laron dwarfism (OMIM **#** 262500), increased responsiveness to growth hormone, and growth hormone insensitivity (OMIM **#** 604271). This gene also acts as a modifier in hypercholesterolemia (OMIM **#** 143890). Patient TNDF182-6 did not show any clinical signs associated with these diseases. A direct causality between the *GHR* gene variants and cognitive impairment has not been clearly established in the literature. Nevertheless, it has been demonstrated that Growth Hormone (GH) receptors are highly expressed in the cerebral regions involved in cognitive function [21]. Furthermore, heterozygous mutations in the *GHR* gene identified in patients with Growth Hormone Insensitivity Syndrome (GHIS) were also correlated with MR in 13.5% of the studied cohort [22].

The *SLC19A3* variant (p.(Asn110Ser)) was found at a homozygous state in TNDF182-6. Her parents were both carriers; however, it was absent in the other three investigated cousins. *SLC19A3* gene has been associated with neuropathies including Thiamine responsive encephalopathy type 2 (OMIM #607483), severe psychomotor retardation and progressive atrophy, Wernicke’s-like encephalopathy (OMIM #606152), and Leigh syndrome (OMIM #256000) [23,24]. All of these clinical entities have been associated with a high incidence of MR. Considering that TNDF182-6 lives abroad, we were unable to perform a cerebral MRI nor a thiamine transport assay to explore encephalopathies related to these diseases.

When viewed in conjunction, (i) the absence of the *GHR* and *SLC19A3* variants in the other affected cousins, (ii) the *in-silico* prediction indicating their deleteriousness as well as (iii) the involvement of both genes in syndromic forms of ID point to their implication in the severe cognitive impairment observed in this patient (TNDF182-6). We hypothesize that *GHR* and *SLC19A3* genes might act either as genetic modifiers that exacerbate the severity of cognitive decline induced by the primary disease-causing mutation in the *MAN2B1* gene or as additional disease loci co-segregating with AM. Based on these results, we recommend that severely affected patients even harbouring the *MAN2B1* gene variants should be investigated at the genomic level or at least at the exome level in order to identify other variants that might account for the additional clinical signs, in particular neurological and cognitive alterations.

It should be noted that it is difficult to differentiate between cognitive impairment secondary to the natural history of the disease and that caused by other genetic aetiologies. However, early diagnosis is important to ensure adequate patient management, to avoid diagnostic errors and to provide efficient genetic counselling.

AM is misdiagnosed in Tunisia and throughout the Arab world [25]. Even in developed countries with high economic income and advanced diagnostic tools, establishing the AM diagnosis often takes a considerable amount of time. A survey conducted in the UK has revealed that many patients were referred to several healthcare specialists before being directed to a metabolic specialist [5]. This was the case for the patients studied by *Beck et al*. for whom the disease diagnosis took a decade or longer in almost half of the cases [15]. Such observations are well illustrated in our report considering that the diagnosis of AM for patient TNDF182-6, who lives in Europe, was never established before its discovery in her affected cousin (TNDF182-3). Complexity of diagnosis is primarily due to the rarity of the disease. Indeed, according to the GnomAD database, the two variants c.685C>T and c.2402dup, identified in the present study within the *MAN2B1* gene, have very low allele frequencies equal to 1.314е-5 and 3.979е-6, respectively. Furthermore, both variants were not found in our in-house control database, nor in the other sample populations represented in GnomAD and GME Variome data, thus highlighting the ultra-rare character of the disease. The misdiagnosis of AM could also be related to the unpredicted progression of the disease course as well as the unawareness of certain healthcare practitioners of the clinical hallmarks of lysosomal storage disorders in general. In our context, the prevalence and incidence of AM in the Tunisian population as well as in other Arab countries is likely to be underestimated given the high consanguinity and endogamy rates characterizing these communities. This is shown by the fourfold higher prevalence of AM, estimated to 1.51/100,000 live births in the Emirati population compared to European countries (Netherlands, Portugal, Czech Republic, and Australia) [25]. Indeed, the high prevalence of intrafamilial marriages, with an inbreeding rate ranging between 25% and 60%, favours the emergence of genetic disorders and raises the frequency of autosomal recessive disorders [26].

Recent reports have shown that HI is often the first AM clinical sign, this was the case for TNDF182-3 patient reported here. Thus, association of hearing loss to motor dysfunctions and cognitive or intellectual disabilities should evoke an AM diagnosis [13,27]. In the absence of adequate biochemical facilities and taking into account the limited resources in genetic screening, if the clinical presentation is consistent with AM, we recommend hotspot mutational screening at first intention for molecular confirmation of the diagnosis. In case of negative results, ES would be of similar cost as the whole *MAN2B1* Sanger sequencing [28]. ES is preferable not only for cost effectiveness, but also to identify other genetic alterations that affect disease phenotype and progression and to provide a more accurate genetic counselling for the affected families.

For patients with AM, shortening the average diagnostic journey by a few years could be the solution to rapid and effective management along with treatment that leads to a longer and healthier life, not only for the patients, but also for their families by reducing the socioeconomic burden of the disease. In another aspect, notable psychological impact has been noticed in AM patients’ families and carers. Caregivers have reported spending between 4 to 24 hours of care for AM patients which often compromises their professional lives [5].

The development of Velmanase alfa ERT is very promising particularly for patients that have not yet developed CNS alterations. This is the case for the three family members: TNDF182-3, 5, and 7. Unfortunately, ERT is not affordable and not yet available in our country. For this reason, allogeneic HSCT has been proposed as a possible treatment option for AM, as for other metabolic diseases, and is suited for our country, especially when considering the prohibitive cost of a lifetime ERT. In addition, HSCT has yielded positive results in Tunisian patients with Gaucher syndrome, another lysosomal storage disease [29].

## Conclusions

AM is widely underdiagnosed, particularly in North African and Middle Eastern countries characterized by a high degree of consanguinity that increases the occurrence of autosomal recessive diseases. Once again, ES has proved to be an effective diagnostic tool that highlights the co-occurrence of genetic variations as causes of complex phenotypes and variable expressivity.

Molecular genetic testing allows early detection of the disease, and consequently, better clinical and therapeutic management as well as accurate genetic counselling. Access to treatment brings great benefits to both patients and their caregivers by alleviating certain severe manifestations, hence positively impacting their quality of life.

## Supporting information

S1 Table Minor allele frequencies of the two *MAN2B1* gene variants. This table comprises the minor alleles frequencies of the two variants c.685C>T and c.2402dup identified in the *MAN2B1* gene, according to the GnomAD database.
(XLSX)

S2 Table Variants identified in hearing loss candidate genes in TNDF182-3 patient. This table includes the list of variants identified in hearing impairment related genes following ES analysis.
(XLSX)

S3 Table List of variants identified in the prioritized genes in TNDF182-6 patient. This table includes the variants potentially associated with cognitive impairment in TNDF182-6 patient.
(XLSX)

S4 Table Biological pathways of *SLC19A3* and *GHR* genes. This table summarizes the biological pathways of *SLC19A3* and *GHR* genes likely associated with cognitive impairment in TNDF182-6 patient.
(DOCX)

S1 File Sanger sequencing validation results of the two variants identified in *GHR* and *SLC19A3* genes. (A) Chromatograms representing the heterozygous status for the two variants c.329A>G, and c.74 T>C located in *GHR* and *SLC19A3* genes, respectively. (B) Chromatograms representing the WT status.
(PPTX)

S2 File Multiple sequence alignments. Results of multiple sequence alignments for the c.329A>G; p.(Asn110Ser) and c.74 T>C; p.(Phe25Ser) variants in *GHR* and *SLC19A3* genes identified in TNDF182-6 patient.
(PPTX)
