# Supplementary material for: Alpha-mannosidosis in Tunisian consanguineous families: Potential involvement of variants in GHR and SLC19A3 genes in the variable expressivity of cognitive impairment
Source: PLoS One. 2021 Oct 6;16(10):e0258202. doi: 10.1371/journal.pone.0258202 (PMC8494324; doi:10.1371/journal.pone.0258202)
Supplement: S4 Table — This table summarizes the biological pathways of SLC19A3 and GHR genes likely associated with cognitive impairment in TNDF182-6 patient. (DOCX) [file pone.0258202.s004.docx]

**S4 Table.** Biological pathways of *SLC19A3* and *GHR* genes potentially associated with cognitive impairment in TNDF182-6 patient

| Genes | Disease (s) (OMIM) | Phenotype(s) MIM number(s) | OMIM gene number | Protein Name (Uniprot) | UniProt Accession Number | Pathways (Reactome, KEGG) | Biological Processes | Sources |
| --- | --- | --- | --- | --- | --- | --- | --- | --- |
| *GHR* | Laron dwarfism | [262500](https://omim.org/entry/262500) | [600946](https://omim.org/entry/600946) | Growth Hormone Receptor | [P10912](https://www.uniprot.org/uniprot/P10912) | Cytokine Signaling in Immune system | activation of Janus kinase activity | UniProtKB, Reactome, GO central, Genecards, BHF-UCL |
|  | Increased responsiveness to growth hormone | [604271](https://omim.org/entry/604271) |  |  |  | Immune System | activation of MAPK activity |  |
|  | Growth hormone insensitivity partial | [604271](https://omim.org/entry/604271) |  |  |  | Growth hormone receptor signaling | cellular response to hormone stimulus |  |
|  | Hypercholesterolemia, familial, (modifier of) | [143890](https://omim.org/entry/143890) |  |  |  | Prolactin receptor signaling | Growth hormone receptor signaling |  |
|  |  |  |  |  |  | Cytokine-cytokine receptor interaction | endocytosis |  |
|  |  |  |  |  |  | Growth hormone synthesis, secretion and action | growth hormone receptor signaling pathway |  |
|  |  |  |  |  |  | JAK-STAT signaling pathway | growth hormone receptor signaling pathway via JAK-STAT |  |
|  |  |  |  |  |  | Neuroactive ligand-receptor interaction | hormone metabolic process |  |
|  |  |  |  |  |  | PI3K-Akt signaling pathway | insulin-like growth factor receptor signaling pathway |  |
|  |  |  |  |  |  |  | positive regulation of multicellular organism growth |  |
|  |  |  |  |  |  |  | positive regulation of peptidyl-tyrosine phosphorylation |  |
|  |  |  |  |  |  |  | positive regulation of receptor signaling pathway via JAK-STAT |  |
|  |  |  |  |  |  |  | positive regulation of tyrosine phosphorylation of STAT protein |  |
|  |  |  |  |  |  |  | receptor internalization |  |
|  |  |  |  |  |  |  | receptor signaling pathway via JAK-STAT |  |
|  |  |  |  |  |  |  | regulation of multicellular organism growth |  |
|  |  |  |  |  |  |  | response to cycloheximide |  |
|  |  |  |  |  |  |  | response to estradiol |  |
| *SLC19A3* | Thiamine metabolism dysfunction syndrome 2 (biotin- or thiamine-responsive encephalopathy type 2) | [607483](https://omim.org/entry/607483) | * 606152 | Thiamine transporter 2 | [Q9BZV2](https://www.uniprot.org/uniprot/Q9BZV2) | Metabolism of vitamins and cofactors | thiamine-containing compound metabolic process |  |
|  |  |  |  |  |  | Metabolism of water-soluble vitamins and cofactors | thiamine transmembrane transport |  |
|  |  |  |  |  |  | Vitamin B1 (thiamin) metabolism | transmembrane transport |  |
|  |  |  |  |  |  | Muscle contraction | regulation of ion transmembrane transport |  |
|  |  |  |  |  |  | Phase 0 - rapid depolarisation | response to toxic substance |  |
|  |  |  |  |  |  |  | sensory perception of pain |  |
|  |  |  |  |  |  |  | sodium ion transmembrane transport |  |
|  |  |  |  |  |  |  | sodium ion transport |  |
