# Supplementary figures and images for: Alpha-mannosidosis in Tunisian consanguineous families: Potential involvement of variants in GHR and SLC19A3 genes in the variable expressivity of cognitive impairment
Source: PLoS One. 2021 Oct 6;16(10):e0258202. doi: 10.1371/journal.pone.0258202 (PMC8494324; doi:10.1371/journal.pone.0258202)

## Slide 1
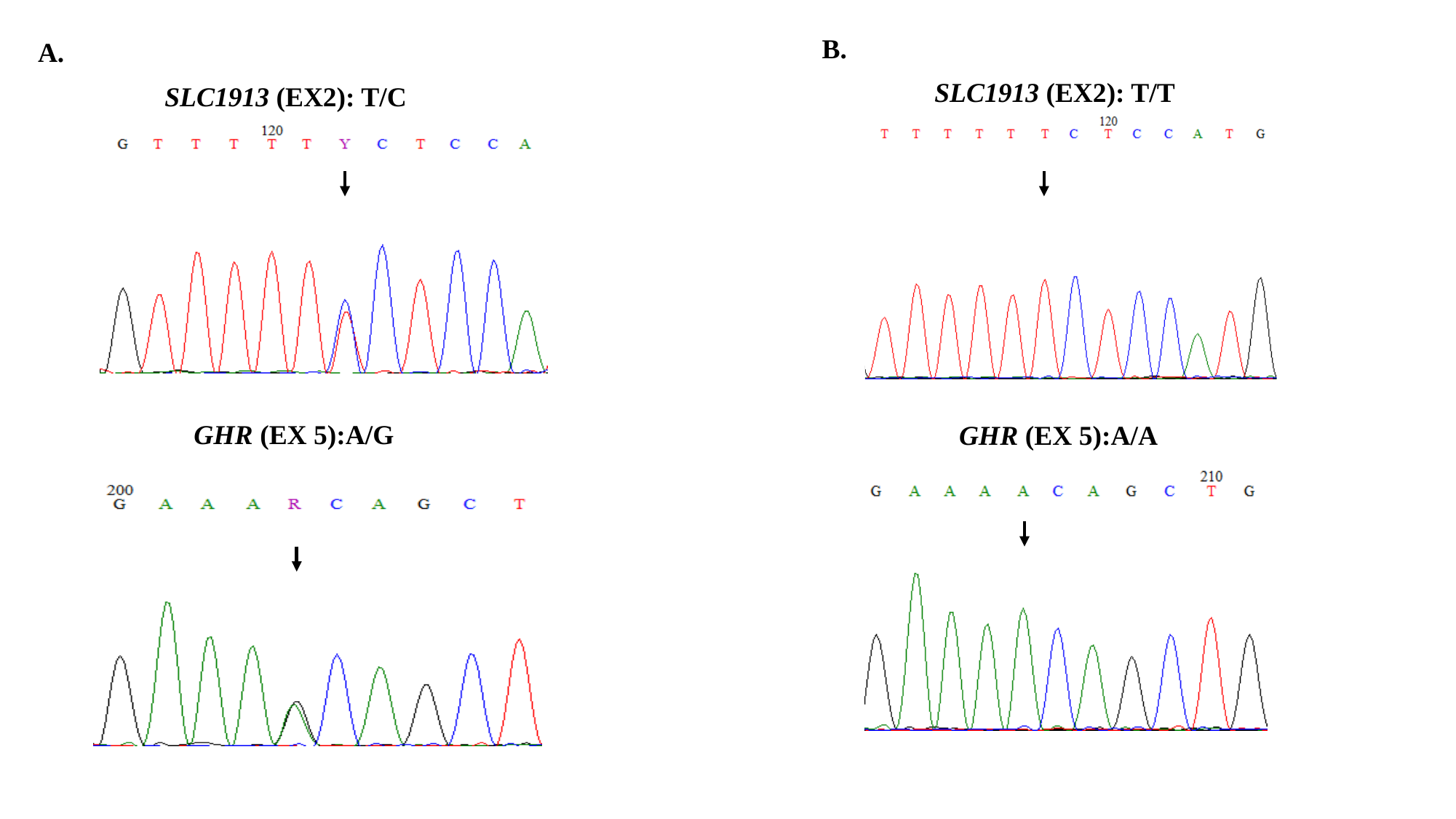

B.
A.
SLC1913 (EX2): T/T
SLC1913 (EX2): T/C
GHR (EX 5):A/G
GHR (EX 5):A/A

Supplement: S1 File — (A) Chromatograms representing the heterozygous status for the two variants c.329A>G, and c.74 T>C located in GHR and SLC19A3 genes, respectively. (B) Chromatograms representing the WT status. (PPTX) [file pone.0258202.s005.pptx]
