# Supplementary material for: Alpha-mannosidosis in Tunisian consanguineous families: Potential involvement of variants in GHR and SLC19A3 genes in the variable expressivity of cognitive impairment
Source: PLoS One. 2021 Oct 6;16(10):e0258202. doi: 10.1371/journal.pone.0258202 (PMC8494324; doi:10.1371/journal.pone.0258202)
Supplement: S2 File — Results of multiple sequence alignments for the c.329A>G; p.(Asn110Ser) and c.74 T>C; p.(Phe25Ser) variants in GHR and SLC19A3 genes identified in TNDF182-6 patient. (PPTX) [file pone.0258202.s006.pptx]

## Slide 1
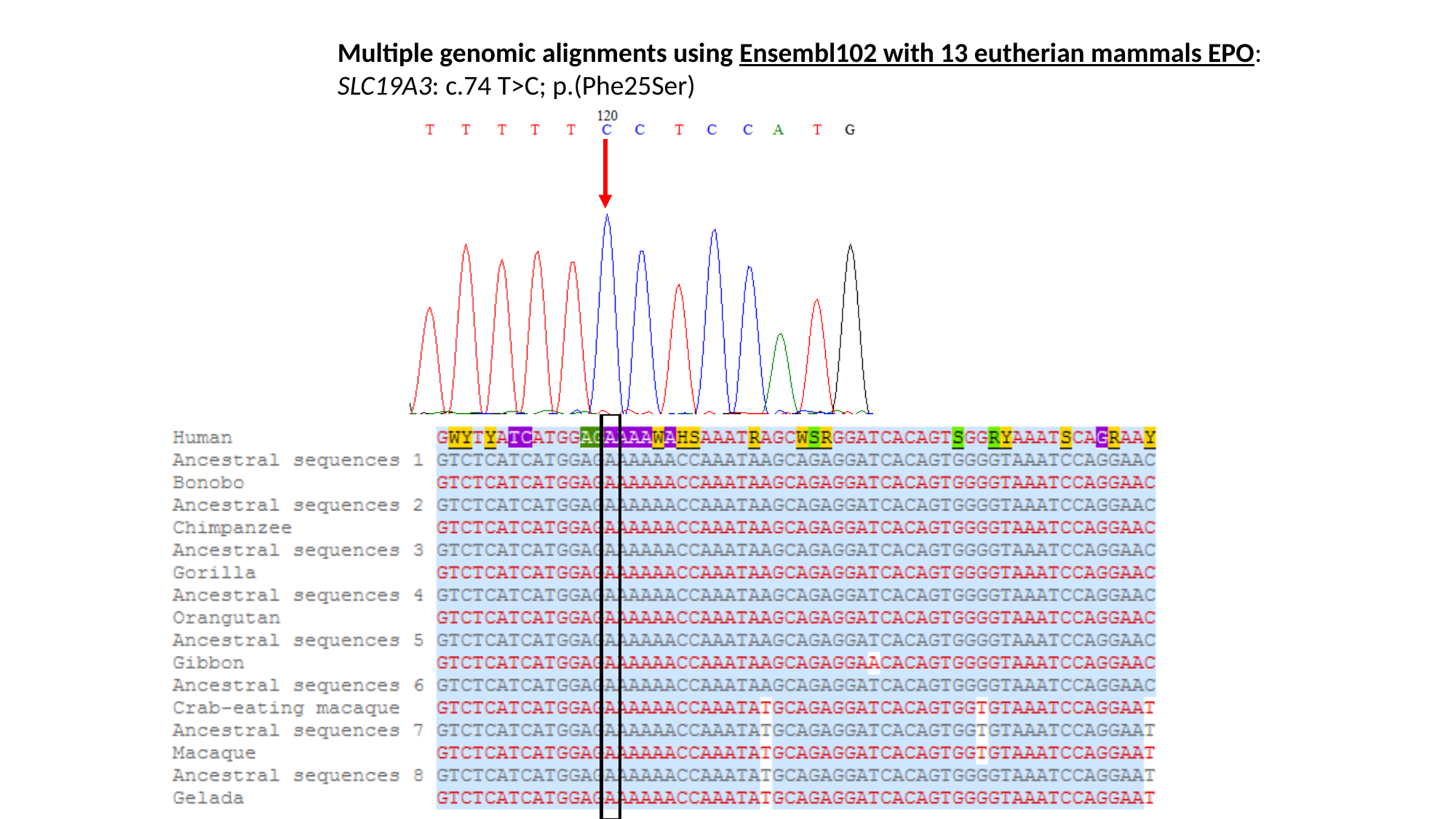

Multiple genomic alignments using Ensembl102 with 13 eutherian mammals EPO:
SLC19A3: c.74 T>C; p.(Phe25Ser)

## Slide 2
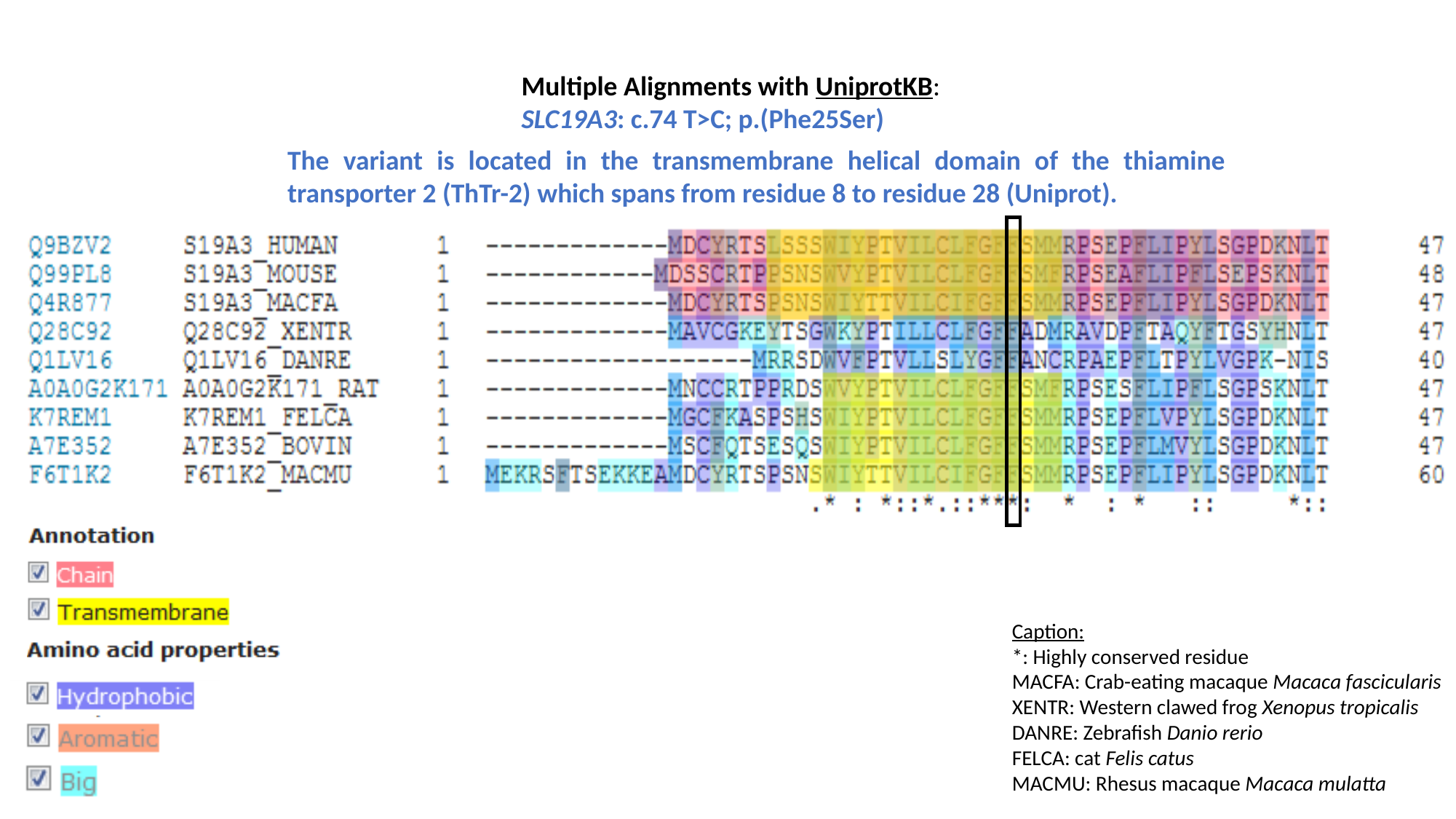

Multiple Alignments with UniprotKB:
SLC19A3: c.74 T>C; p.(Phe25Ser)
The variant is located in the transmembrane helical domain of the thiamine transporter 2 (ThTr-2) which spans from residue 8 to residue 28 (Uniprot).
Caption:
*: Highly conserved residue
MACFA: Crab-eating macaque Macaca fascicularis
XENTR: Western clawed frog Xenopus tropicalis
DANRE: Zebrafish Danio rerio
FELCA: cat Felis catus
MACMU: Rhesus macaque Macaca mulatta

## Slide 3
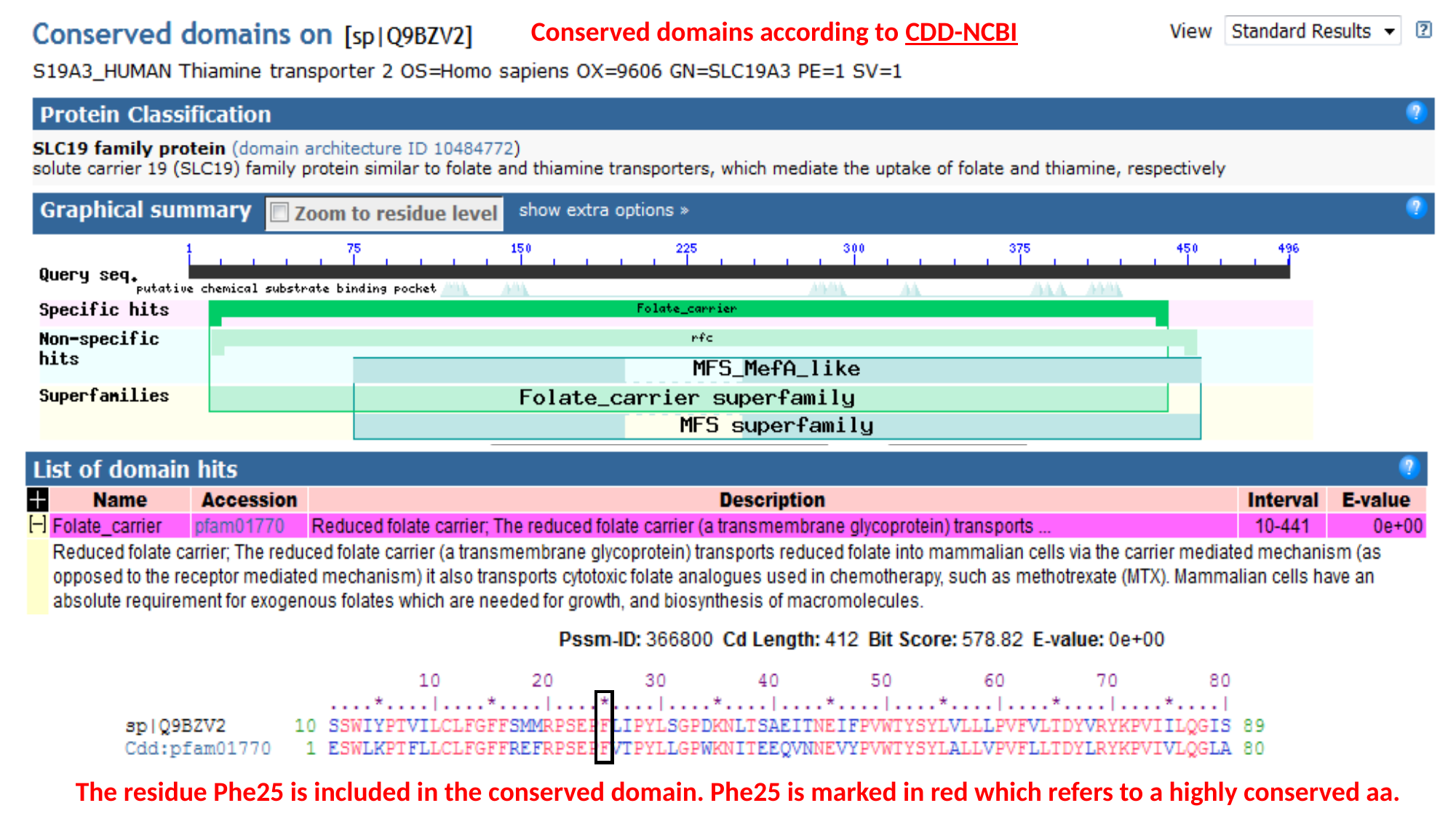

Conserved domains according to CDD-NCBI
The residue Phe25 is included in the conserved domain. Phe25 is marked in red which refers to a highly conserved aa.

## Slide 4
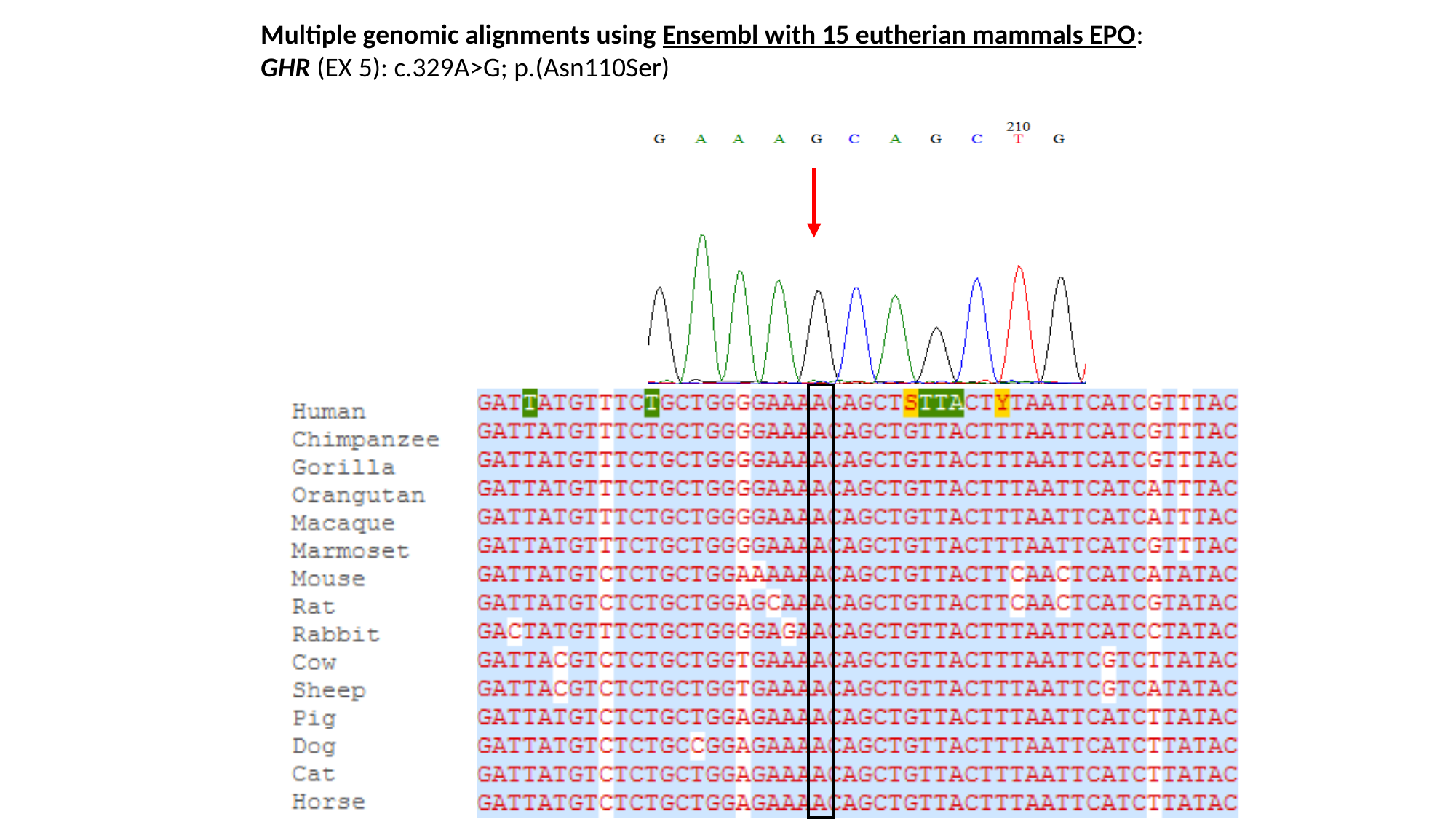

Multiple genomic alignments using Ensembl with 15 eutherian mammals EPO:
GHR (EX 5): c.329A>G; p.(Asn110Ser)

## Slide 5
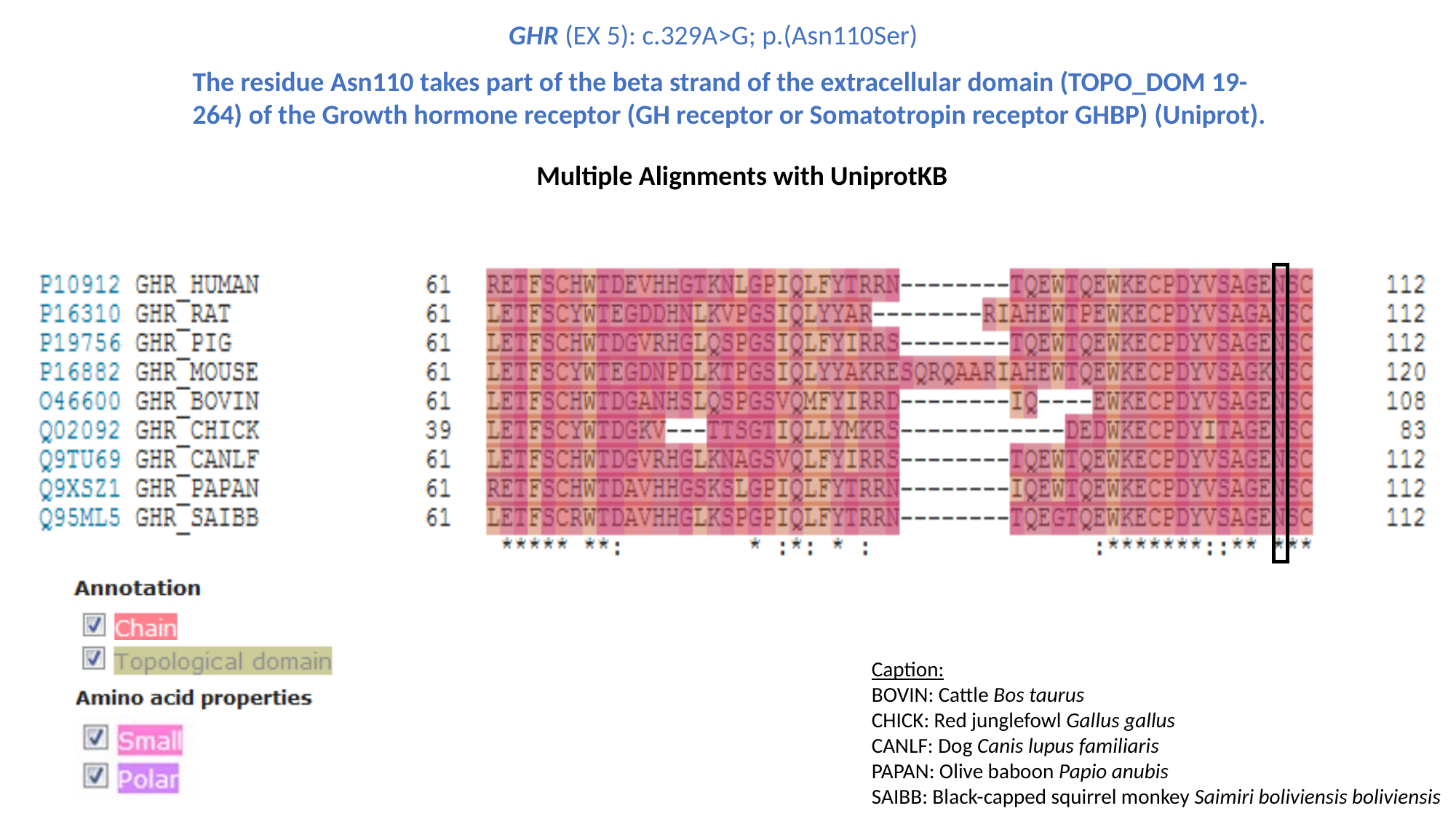

GHR (EX 5): c.329A>G; p.(Asn110Ser)
The residue Asn110 takes part of the beta strand of the extracellular domain (TOPO_DOM 19-264) of the Growth hormone receptor (GH receptor or Somatotropin receptor GHBP) (Uniprot).
Multiple Alignments with UniprotKB
Caption:
BOVIN: Cattle Bos taurus
CHICK: Red junglefowl Gallus gallus
CANLF: Dog Canis lupus familiaris
PAPAN: Olive baboon Papio anubis
SAIBB: Black-capped squirrel monkey Saimiri boliviensis boliviensis

## Slide 6
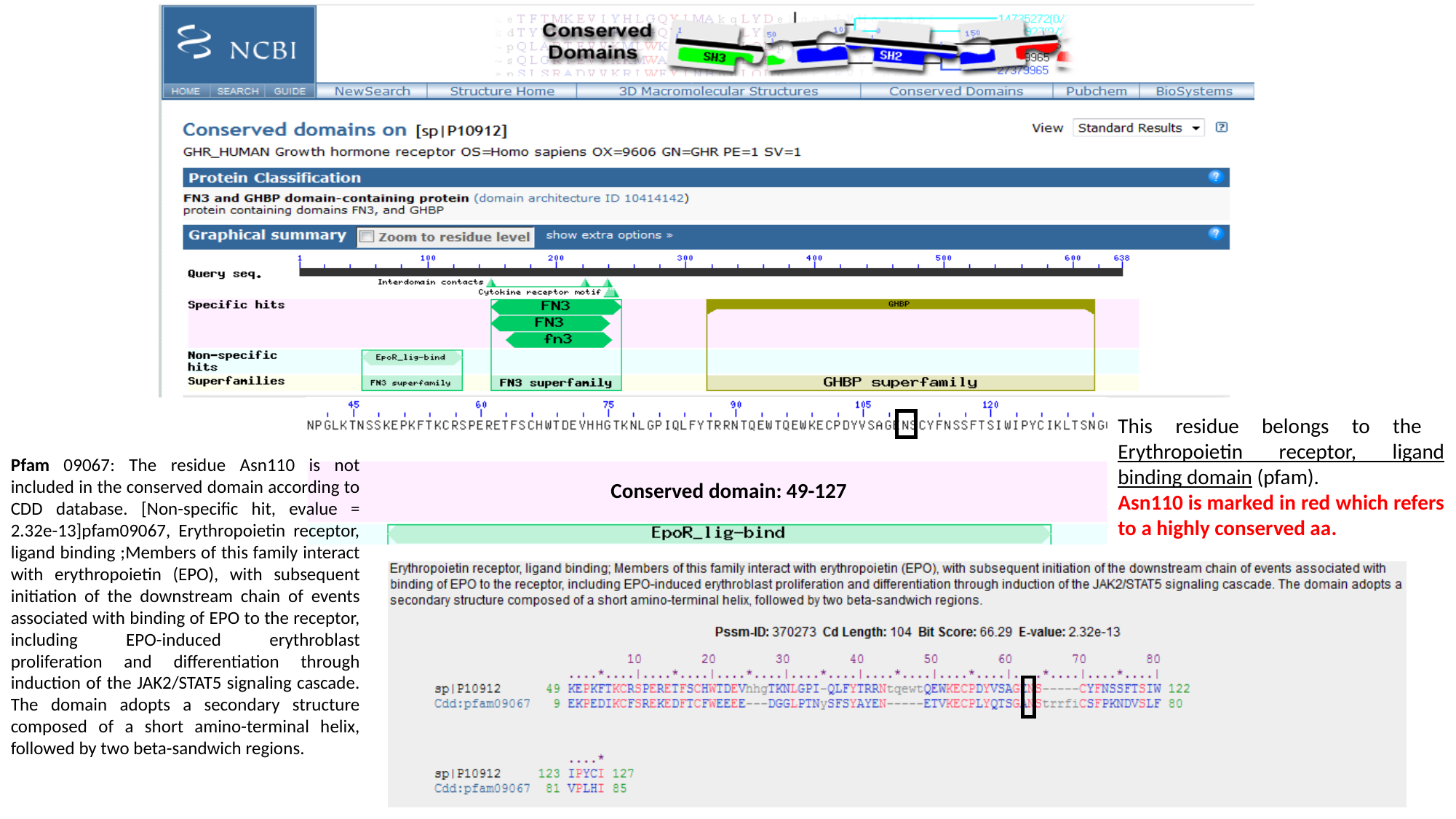

This residue belongs to the Erythropoietin receptor, ligand binding domain (pfam).
Asn110 is marked in red which refers to a highly conserved aa.
Pfam 09067: The residue Asn110 is not included in the conserved domain according to CDD database. [Non-specific hit, evalue = 2.32e-13]pfam09067, Erythropoietin receptor, ligand binding ;Members of this family interact with erythropoietin (EPO), with subsequent initiation of the downstream chain of events associated with binding of EPO to the receptor, including EPO-induced erythroblast proliferation and differentiation through induction of the JAK2/STAT5 signaling cascade. The domain adopts a secondary structure composed of a short amino-terminal helix, followed by two beta-sandwich regions.
Conserved domain: 49-127
